# Characterization of *N*-Acyl Homoserine Lactones in *Vibrio tasmaniensis* LGP32 by a Biosensor-Based UHPLC-HRMS/MS Method

**DOI:** 10.3390/s17040906

**Published:** 2017-04-20

**Authors:** Léa Girard, Élodie Blanchet, Laurent Intertaglia, Julia Baudart, Didier Stien, Marcelino Suzuki, Philippe Lebaron, Raphaël Lami

**Affiliations:** 1 Sorbonne Universités, UPMC Univ Paris 6, CNRS, Laboratoire de Biodiversité et Biotechnologies Microbiennes (LBBM), Observatoire Océanologique, F-66650 Banyuls/Mer, France; girard@obs-banyuls.fr (L.G.); blanchet.elodie1@gmail.com (É.B.); baudart@obs-banyuls.fr (J.B.); didier.stien@cnrs.fr (D.S.); suzuki@obs-banyuls.fr (M.S.); lebaron@obs-banyuls.fr (P.L.); 2 Sorbonne Universités, UPMC Univ Paris 06, CNRS, Observatoire Océanologique de Banyuls (OOB), F-66650 Banyuls/Mer, France; intertaglia@obs-banyuls.fr

**Keywords:** quorum sensing, *N*-acyl-homoserine lactone (AHL), *Vibrio tasmaniensis* LGP32, fractionation, biosensors, UHPLC-HRMS/MS

## Abstract

Since the discovery of quorum sensing (QS) in the 1970s, many studies have demonstrated that *Vibrio* species coordinate activities such as biofilm formation, virulence, pathogenesis, and bioluminescence, through a large group of molecules called *N*-acyl homoserine lactones (AHLs). However, despite the extensive knowledge on the involved molecules and the biological processes controlled by QS in a few selected *Vibrio* strains, less is known about the overall diversity of AHLs produced by a broader range of environmental strains. To investigate the prevalence of QS capability of *Vibrio* environmental strains we analyzed 87 *Vibrio* spp. strains from the Banyuls Bacterial Culture Collection (WDCM911) for their ability to produce AHLs. This screening was based on three biosensors, which cover a large spectrum of AHLs, and revealed that only 9% of the screened isolates produced AHLs in the defined experimental conditions. Among these AHL-producing strains, *Vibrio tasmaniensis* LGP32 is a well-known pathogen of bivalves. We further analyzed the diversity of AHLs produced by this strain using a sensitive bioguided UHPLC-HRMS/MS approach (Ultra-High-Performance Liquid Chromatography followed by High-Resolution tandem Mass Spectrometry) and we identified C10-HSL, OH-C12-HSL, oxo-C12-HSL and C14:1-HSL as QS molecules. This is the first report that documents the production of AHL by *Vibrio tasmaniensis* LGP32.

## 1. Introduction

Bacteria of the genus *Vibrio* are ubiquitous marine bacteria belonging to the *Gammaproteobacteria* class and this genus includes both non-pathogenic and pathogenic species. Among the 133 described species of *Vibrio* (www.bacterio.net), at least 12 (e.g., *V. cholerae, V. vulnificus, V. parahaemolyticus*) are well known to be highly pathogenic to humans and a large number of other strains are pathogens of a wide range of marine organisms [1,2,3]. These opportunistic bacteria are also able to colonize diverse substrates, to participate in biofilm production [4,5], and to associate with phytoplankton, zooplankton, marine vertebrates and invertebrates [6,7,8,9]. Quorum sensing (QS) has been shown to be involved in many of these processes and associations [10,11], whereby the population density regulates gene expression, through the emission of self-generated small signal molecules named autoinducers (AI), to obtain a concerted physiological response [12,13,14].

Type 1 autoinducers (AI-1) also called *N*-acyl homoserine lactones (AHLs) are signaling compounds used by many bacteria for communication among single or closely related species [15,16,17,18]. In Gram-negative bacteria, the AHLs are synthesized by one or more synthases whose LuxI and LuxM/AinS are the most widespread [19,20,21]. Since the first investigations on QS in the symbiotic and bioluminescent *V. fischeri*, other *Vibrio* species were shown to communicate using AHLs [22,23,24]. QS molecules and signaling pathways are well known for *V. cholerae*, *V. fischeri*, *V. harveyi*, *V. vulnificus* and *V. anguillarum* as are their roles in bioluminescence, pathogenicity and biofilm formation [25]. Nonetheless, the prevalence and diversity of AHL-based QS in other environmental strains of *Vibrio* remains poorly understood.

Direct in situ studies of AHLs production in the marine environment have been limited and hampered by the fact that AHLs are likely released locally in microenvironments of high cellular concentration and thus at low total concentrations in a water sample. Therefore, quantifying AHL production directly in the environment is extremely difficult [26]. To overcome this problem, the isolation and identification of environmental strains able to produce AHLs and the characterization of these compounds appear as the best alternative. For these purpose, several detection approaches have been previously developed and most of them are using bioreporters able to detect a wide range of AHLs [27,28,29]. Most of these bioreporters are genetically modified strains, where reporter genes such as violacein or phenazine production, β-galactosidase or green fluorescent protein (GFP), are under the control of AHL-based-quorum sensing inducing promoters [29,30]. The use of multiple reporter strains does not allow the characterization of individual AHL molecules but provides an activation pattern reflecting a specific AHLs production phenotype in the defined culture condition [22,31]. Thus, in the past 10 years, numerous studies characterized AHLs—in combination or not with reporters—using analytical chemistry tools. The characterization of known AHLs is most commonly achieved by thin-layer chromatography (TLC) or High Performance Liquid Chromatography (HPLC) and comparison to standards [31,32,33,34]. However, since the diversity of AHLs is far from completely described [35], the characterization of *novel* AHLs requires the combination of chromatography with structural analysis, like Gas Chromatography coupled with Mass Spectrometry (GC-MS; [36]), HPLC coupled with tandem Mass Spectrometry (HPLC-MS/MS) or Nuclear Magnetic Resonance (NMR) [37]. The recent development of more sensitive and accurate techniques such as Ultra-High Performance Liquid Chromatography (UHPLC) combined to High Resolution Mass Spectrometry (HRMS) have yielded a high diversity of *novel* AHL molecules [38,39].

*Vibrio tasmaniensis* strain LGP32, previously named *Vibrio splendidus*, has been widely used as a model organism for the study of host-pathogens relationship in bivalves [40,41,42]. This strain had been isolated from diseased oysters during mortality events in France and is a facultative intracellular pathogen that attach and invade oyster hemocytes [43]. The complete genome analysis of LGP32 has revealed that—like other pathogenic ones, such as *V. harveyi*—this strain, harbors QS systems based on three different autoinducers (AHL, CAI-1 and AI-2) [16]. Interestingly, De Decker et al. showed in 2013 a possible involvement of QS in the virulence mechanisms of LGP32 [44], but remarkably the AHL molecules associated with this strain have not yet been characterized.

The aim of this study was to first evaluate the AHL production among environmental *Vibrio* spp. strains isolated from a large diversity of marine environments. The result of this screening led us to focus on the chemical diversity of AHLs in *Vibrio tasmaniensis* LGP32 using an optimized protocol to potentially maximize the discovery of *novel* AHLs. This protocol includes the use of large extraction volumes (3 L) followed by a biosensor-based screening of HPLC fractions prior to structural characterization by UHPLC-HRMS/MS. In this study, we also assessed the limit of detection of various approaches and extended the range of tested AHLs compared to previous studies. Our methodological approach allowed, for the first time, the characterization of AHL compounds involved in the QS of the important bivalve pathogen *Vibrio tasmaniensis* LGP32.

## 2. Materials and Methods

### 2.1. Culture Collection and Strains Identification

The Banyuls Bacterial Culture Collection (BBCC) is referenced in the World Data Center for Microorganisms as WDCM911 and harbors more than 2000 bacterial strains isolated from different geographical sites which are mostly heterotrophic marine bacteria identified on the 16S rRNA gene sequence [45]. Genomic DNA of each of the strains was extracted with the Wizard Genomic DNA purification kit (Promega, Charbonnières-les-Bains, France) as previously described [46]. PCR was performed using the universal primers targeting bacteria, 27Fmod (AGRGTTTGATC-MTGGCTCAG) [47] and 1492Rmod (TACGGYTACCTTGTTAYGACTT) [48]. PCR products were purified using the Agencourt AMPureXp purification kit (Beckman Coulter, Villepinte, France) and sequenced as described previously with an AB3130xl genetic analyzer (Applied Biosystems, Courtaboeuf, France). All molecular biology instrumentation was available through the Bio2Mar platform at the Observatoire Oceanologique de Banyuls-sur-Mer. The 16S rRNA gene sequences were compared to sequences within the NCBI nt database using the Basic Local Alignment Search Tool—2 sequences [49,50]. For the QS screening we selected all strains in the BBCC Culture Collection with a similarity percent above 98% to known *Vibrio* species (i.e., Table 1). The List of the 87 tested strains with their origin, their identification by 16S rRNA gene sequence and their GenBank accession numbers can be found in Table S1 of the Supplementary Material.

### 2.2. Biosensor Assays

The detection of AHLs in culture supernatants and HPLC fractions (see details in Section 2.3) followed previously described protocols using the biosensors *Pseudomonas putida (P. putida)*, *Escherichia coli (E. coli)* and *Chromobacterium violaceum (C. violaceum)*. The biosensors *Pseudomonas putida* F117 (pRK-C12; Kmr; *ppuI::npt*) and *Escherichia coli* MT102 (pJBA132) were used for the detection of AHLs in liquid medium and *Chromobacterium violaceum* CV026 was used for the detection of AHLs in solid medium [51,52,53]. Briefly, 50 µL of culture supernatants, obtained by centrifugation of 2 mL of culture in Marine Broth (MB), grown overnight at 25 °C under shaking (100 RPM), and 20 µL of HPLC fractions at 10 mg·mL^−1^ in dimethyl sulfoxide (DMSO) diluted in Luria Bertani (LB) broth (1/4 *v*:*v*), were tested in triplicate. For the biosensors *E. coli* and *P. putida*, microplates were incubated respectively at 30 °C and 37 °C and OD was measured at 535 nm after 0, 5 and 24 h of incubation. OD620 was also measured to control for biosensor cell growth [30,53,54,55]. For the biosensor *C. violaceum*, culture plates were incubated at 30 °C and purple zones of violacein production were inspected after 24 h [51]. For all tests, negative controls consisted of biosensor cultures without supernatant or fractions, and sterile LB medium. Biosensor cultures with addition of commercial AHLs (C6-HSL and oxo-C10-HSL, Cayman Chemical, Ann Arbor, MI, USA) were used as positive controls. To determine the AHL concentration range of detection of each biosensor, a dilution range from 0.01 to 25,000 nM of 27 commercial AHL standards (Cayman Chemical, see Table 2) was tested following the protocols described above.

### 2.3. AHL Extraction and HPLC Fractionation of LGP32

LGP32 was cultured in 3 L of Marine Broth (Difco, Le pont de Claix, France) at 25 °C under shaking (100 RPM) for 24 h (representing a late exponential phase, pH 7.5). A liquid-liquid extraction of the culture was performed with ethyl acetate (1/3 *v*:*v*) in a separatory funnel. The organic phase was evaporated to dryness and the extract was re-suspended in 1 mL of HPLC grade DMSO. The extract was fractionated using a separative HPLC system with two Varian Prep Star pumps, a manual injector, a Dionex Ultimate 3000 RS variable wavelength detector and a Dionex Ultimate 3000 fraction collector (Thermo Scientific, Courtaboeuf, France). The column was a Phenomenex Luna C18 (21.2 mm × 250 mm), with 5 μm particle size, and the flow rate was set to 20 mL·min^−1^. The mobile phase consisted of HPLC grade H_2_O and CH_3_CN at different proportions starting at 70:30 for 3 min, followed by a 12 min linear gradient from 70:30 to 0:100, followed by 100% CH_3_CN for 10 min. 22 fractions were collected every minute between 3 and 25 min. The solvent was removed with a HT-4X system (Genevac, Biopharma Technologies France, Lyon, France), each fraction was dissolved in 100 μL DMSO and diluted at 1/4 with LB medium (*v*/*v*) to perform the biosensor tests. Positive fractions were further analyzed by UHPLC-HRMS/MS. pH was controlled at each step of our experimental process and maintained in between 6 and 7.

### 2.4. AHL Detection by UHPLC-HRMS/MS

Prior to injection, fractions were diluted at 1 mg·mL^−1^ and 5 µL were injected. UHPLC-MS analyses were performed with a Dionex Ultimate 3000 UHPLC-HESI HRMS Q-Exactive focus system (Thermo Scientific) controlled by the Xcalibur software. The column was a Hypersil GOLD C18 (2.1 mm × 150 mm) with 1.9 µm particle size (Thermo Scientific). The column oven was set to 50 °C. The flow rate was maintained at 0.8 mL·min^−1^. The mobile phase was composed of 0.1% formic acid in water (eluent A) and 0.1% formic acid in acetonitrile (B). A gradient profile was used, starting with 100% of A, and keeping this composition constant for 5 min. The proportion of B was linearly increased to 100% in 5 min, and was left at 100% for 5 min. Settings for the ion source were: 20 aux gas flow rate, 75 sheath gas flow rate, 4 μA spray current, 3 kV spray voltage, 350 °C capillary temperature, 450 °C heater temperature, and 40 S-lens RF and nitrogen was used as nebulizing gas by the ion trap source.

Firstly, MS and MS/MS profiles were recorded alternating between a full scan (scan range 130 to 900 *m*/*z*) and All Ion Fragmentation (AIF) mode [scan range 60 to 600 *m*/*z*, normalized-collision energy (NCE) 25] to determine molecular weights and identify chromatographic peaks generating fragment ions at *m*/*z* 102.0555. Mass resolution was set at 35,000, AGC target was 1 × 10^6^ and 5 × 10^4^ respectively for the full scan and AIF mode, and injection time was 40 ms. The study of mass spectra obtained for our standard molecules and the study of Patel et al. [35] reveal that fragment ions at *m*/*z* 102.055, 84.045, 74.06 and 56.05 are specific of the homoserine lactone (HSL) moiety and these signals were chosen as the specific ions indicating the presence of AHL-type compounds. In a second step, we used a SIM (resolution 35,000) and dd-ms2 (resolution 17,500) mode to confirm the AHLs identification. MS/MS scans were isolated using an isolation width of 3.0 Da, fragmentations were performed at 17,500 with a collision energy of 20 eV. The limit of detection (LOD) of our UHPLC-HRMS method was established based on the standard deviation of the response to the 27 AHLs at 50 nmol·L^−1^ and the slope of a 10-fold concentration range (*R*^2^ values > 0.99). Briefly, a calibration curve was constructed using a simple linear regression analysis from the injection of AHL standard solution mixtures at concentration ranging from 5 to 500 nmol·L^−1^. Each AHL was injected 10 times at 50 nmol·L^−1^ and LOD was expressed as 3.3 times the standard deviation (LOD = 3.3SD).

## 3. Results

### 3.1. Biosensors Strains & UHPLC-HRMS/MS AHL Detection Limits

To define the specificity and the sensitivity of the three biosensors strains, we evaluated their responses to 27 commercially available AHL molecules (Table 2). The *C. violaceum* biosensor (CV026) showed a highly specific response to short chain AHLs (<10 carbons in the acyl chain) with a high sensitivity for C6-HSL (2.5 nmol·L^−1^), C7-HSL (1 nmol·L^−1^) and OH-C10-HSL (2.5 nmol·L^−1^). The biosensors *E. coli* MT102 detected mostly short acyl side chain AHLs and *P. putida* F117 showed the lowest specificity, by detecting 23 of 27 AHLs, while exhibited highest sensitivity to 3-oxo-HSL (LOD < 0.001 nmol·L^−1^; Table 2). Overall, GFP-based biosensors, MT102 and F117, showed the highest sensitivity levels to the most tested molecules. Finally, C4-HSL was only detected by *C. violaceum*.

Considering the overlap between the biosensors CV026 and MT102 and the suitability of GFP-based biosensors for high-throughput analyses, our fractionation and structural determination by UHPLC-HRMS/MS was solely based on detection by *E. coli* MT102 and *P. putida* F117 as biosensors. We evaluated the LOD of our UHPLC/HRMS-MS devices for 27 commercially available AHLs also used as AHL standards in our study and these detection limits were between 2.39 nmol·L^−1^ for the 3-OH-C12 HSL and 36.58 nmol·L^−1^ for the 3-OH-C14 HSL. Overall, short chain AHLs (with the notable exception of C4-HSL) presented lower LODs compared to long acyl side chain AHLs. All MS/MS fragmentation spectra of the 27 AHL standards can be found in Figure S1 of the Supplementary Material.

### 3.2. Screening of AHL-Producing Vibrio Strains

A total of 87 *Vibrio* spp. strains were selected from the BBCC Culture Collection of c.a. 2000 strains. The strains were previously isolated from seawater, crater lake water, sea urchin, green alga, sponge, Rodophyta, macrophytes, Urochordata or jellyfish and from different geographical sites. These strains are closely related to 28 described species, as shown in Table 1. Isolates closely related to the Splendidus clade represented 40% of the strains, the highest number of isolates was related to *V. gigantis* (16 isolates) mostly isolated from benthic and pelagic macro-organisms followed by *V. splendidus* (8 isolates) which were rather isolated from seawater. The 26 remaining species varied between 1 and 7 isolates. A 16S rRNA phylogenetic analysis of the strains and the GenBank accession numbers are provided in the Supplementary Material Figure S2 and Table S1.

In order to detect a broad diversity of AHLs, the isolates were tested for their capability to produce AHLs using three biosensors, *P. putida* F117 (pKR-C12), *E. coli* MT102 (pJBA-132) and *C. violaceum* (CV026; Table 1). The vast majority of the analyzed isolates (91%) did not produced AHLs able to activate any of our three biosensors in the defined experimental conditions. Eight isolates (9%) closely related to the species *V. mytili, V. metschnikovii, V. scophtalmi, V. tasmaniensis and V. ordalii* activated at least one of the three bioreporter strains. Strain BBCC 1015 (*V. ordalii*), CIP 107715 (*V. tasmaniensis,* LGP32) and BBCC 2428 (*V. mytili*) activated the biosensor *C. violaceum* CV026. The strain BBCC 1015 (*V. ordalii*), BBCC 1026 and 1055 (*V. metschnikovii*), BBCC 1228, 1237, and 1238 (*V. scophtalmi*), CIP 107715 (*V. tasmaniensis*, LGP32) activated the biosensor *E. coli* MT102. All these strains, except for LGP32, also activated the biosensor *P. putida* F117. In addition to the fact that LGP32 is likely to produce a diverse panel of AHLs, this strain is also a well-known pathogen of marine invertebrates including some with commercial interest [1]. It therefore appeared important to describe the AHLs produced by this strain.

### 3.3. Vibrio Tasmaniensis LGP32: AHLs Characterization by UHPLC-HRMS/MS

The culture supernatant was extracted with ethyl acetate and fractionated into 22 fractions, identified as LGP32_a to LGP32_v. These fractions were then tested for AHL production using the biosensor strains *E. coli* MT102 and *P. putida* F117. A total of four fractions (LGP32_l, LGP32_m, LGP32_o and LGP32_p) were positive with at least one of the biosensors. UHPLC-HRMS and UHPLC-HRMS/MS analyses were then performed to identify AHLs. The presence of AHLs in the fractions were revealed by the detection in the fragmentation patterns of at least one of the following characteristic lactone ring fragments (*m*/*z* 102.055, 84.045, 74.061 and 56.050) [35]. In the condition tested, four different AHLs were detected including unsubstituted, oxo and hydroxy AHLs at the third carbon atom (Table 3). The AHLs detected for this strain were C10-HSL (*N*-decanoyl homoserine lactone), OH-C12-HSL (*N*-3-hydroxy-dodecanoyl homoserine lactone), oxo-C12-HSL (*N*-3-oxo-dodecanoyl homoserine lactone) and C14:1-HSL (*N*-tetradecenoyl homoserine lactone). These identifications were supported by the analysis of 27 standard AHLs (Table 4), the retention time and the exact mass of the [M + H]^+^ pseudo-molecular ion (precision 3 ppm). However, the exact double bond position on the acyl side chain of the C14:1-HSL has not been determined as it is not easily achievable by mass spectroscopy and such determination would have required derivatization methods or high-field NMR.

## 4. Discussion

### 4.1. Diversity of AHL Producing Strains

We investigated AHL production among 87 *Vibrio* spp. strains using three different bioreporter strains. Remarkably, while the production of AHL quorum-sensing signal molecules has been widely reported among *Vibrio*, only a small percentage of our *Vibrio* spp. strains (9%) were shown to produce AHLs.

This result was different from the observations made by Garcia-Aljaro [22] and Purohit et al., [56] who found that the majority (85%) of *Vibrio* spp. strains in their collection were AHL producers. Similarly, Yang et al. focused on 25 strains and found 23 positive for AHL production [31]. By contrast, and in line with our study, Rasmussen et al., found only 32 positive strains (10%) among the 301 tested in their culture collection [24]. Different non-exclusive hypotheses can be made to explain this low percentage of AHL producing *Vibrio*: (1) the growth conditions might not have been optimal for all *Vibrio* spp. strains to reach the necessary density to produce AHLs or the threshold is variable among strains [57]; (2) The concentrations of produced AHLs might be below the limit of detection of our biosensors; (3) The strains produce *novel* or undetected AHLs, that are not activating our biosensors and (4) The strains do not contain the machinery necessary to produce AHLs. Since we followed the culture growth and we did not observe significant differences in OD between positive and negative strains, hypothesis (1) is somewhat less supported by our results than the remainder explanations. The differences between the activation profiles among strains reflect a diversity of produced AHLs and that can be affected by the genetic diversity of AHL synthases but also the presence of one or more synthases in their genome [58], in agreement with previous observations showing high intraspecific genetic diversity in the genus *Vibrio* [59]. However, interactions and ecological processes that drive or are affected by this phenotypic variation are still poorly understood and warrant further studies [60,61].

### 4.2. AHL Diversity of Vibrio Tasmaniensis LGP32

The observation of AHL production in *Vibrio tasmaniensis* LGP32 is notable, as this strain is a well-known pathogen and producer of outer membrane vesicles (OMVs), OmpU, porins and metalloproteases [41,62,63], all involved in the virulence in oyster larvae [44]. We detected four produced AHLs: C10-HSL, 3-OH-C12-HSL, 3-oxo-C12-HSL and C14:1-HSL and this is the first report of C10-HSL, 3-oxo-C12-HSL and C14:1-HSL in *Vibrio* strains belonging to the Splendidus clade. On the other hand 3-OH-C12-HSL have already been reported [24,54]. Among these four AHLs, three have already been identified in the putative pathogens *V. campbellii*, *V. furnissii*, *V. fluvialis and V. anguillarum* [32,64,65,66]. More broadly among Gram-negative *Proteobacteria*, 3-oxo-C12-HSL controls biofilm production in *Pseudomonas aeruginosa* and has a determining immunomodulatory activity of the human host [67], 3-OH-C12-HSL is involved in virulence factor production by *Acinetobacter baumannii* [68], and finally C14:1-HSL participate in the establishment of a necrosis phenotype in *Agrobacterium vitis* [69]. Considering the AHLs regulation of virulence mechanisms in other *Vibrio* species [11,70,71] and other *Proteobacteria*, our results might add to the understanding of the role of AHLs in the physiology and the pathogenicity of these microorganisms.

### 4.3. Method Performance

In addition to the results described above, our work also provides data on AHL detection limits by AHL bioreporter strains and by UHPLC-HRMS/MS. Such data, and more especially their comparison, is crucial for future studies of AHL production by *Proteobacteria*. Previous reports have already characterized the limits of detection for these biosensors [36,51,53]. However, in this study, we extended our work to a larger panel of AHLs including long acyl side chain compounds (>14 carbons in the acyl chain), that have not been determined before. While numerous studies have demonstrated that the ability to detect OH-HSL is unique to *Agrobacterium tumefaciens* NTL4 (pZLR4) [22,72], we showed that *Pseudomonas putida* F117 is also able to detect that type of AHLs with similar detection limits (between 13.43 and 0.125 nmol·L^−1^).

Our UHPLC-HRMS protocol yielded a good separation and well defined peaks for 26 AHL standards, with a mass accuracy for all standards below 3 ppm and a median LOD of 10.58 nmol·L^−1^. Finally, the combination of two GFP-based biosensors, *E. coli* MT102 and *P. putida* F117, was responsive to over 90% of the AHL standards and exhibited a lower limit of detection than analytical UHPLC-HRMS methods [24,35]. Our protocol included larger culture volumes compared to those used in similar studies (75 µL to 50 mL [24,35,56]), an activity-based screening of HPLC fractions, and UHPLC-HRMS/MS structural determination that can in theory increase the detection of rarer and/or *novel* AHL structures when compared with these previously published approaches. The simple fact that HPLC fractions are less complex than supernatants or raw extracts might significantly increase the discovery of novel QS-receptor agonists in the future. In the current study, we did not quantify the AHLs production, but this method is fully compatible with quantification.

The non-targeted UHPLC/HRMS/MS method reported here allows, prior to NMR structure determination, the identification of *novel* AHLs without any corresponding standards. The AHL identification is based on the study of MS/MS fragmentation patterns and the search of characteristic fragment ions corresponding to the lactone ring fragmentation [35]. In theory, this method could be more suitable for comparative studies of AHL production. Unfortunately, we did not uncover *novel* AHLs in LGP32 to demonstrate this potential but several ongoing studies with other gram-negative bacterial strains in our group have already yielded *novel* AHL structures using the same approach.

## 5. Conclusions

In this work, we confirmed that AHL are produced by different species of *Vibrio* and that this production varies among different strains of the same species, pointing to the need of further studies to understand the biological origin as well as ecological significance of this intraspecific variation. To our knowledge, this is the first study that demonstrated AHLs production of *Vibrio tasmaniensis* LGP32 a pathogenic bacterium involved in oyster mortality. Four AHLs, namely C10-HSL, OH-C12-HSL, oxo-C12-HSL and C14:1-HSL were detected and identified by our novel approach combining a HPLC fractionation followed by an activity-based AHL identification by UHPLC-HRMS/MS. This study should be useful for the understanding of the virulence and physiology mechanisms of LGP32. In addition, the limit of detection of a large panel of AHL standards was established and a broader response range was highlighted for the biosensors *Escherichia coli* MT102 and *Pseudomonas putida* F117 compared to previous studies.

## Figures and Tables

**Table 1:** Activation patterns of *Vibrio* species when tested against three biosensor strains: F117, *Pseudomonas putida* (pKR-C12); MT102, *Escherichia coli* (pJBA-132) and CV026, *Chromobacterium violaceum*.

Closest Relative Species	Number of Isolates	BBCC Code	CV026	MT102	F117
***Vibrio atlanticus***	*1*	2313	-	-	-
***Vibrio brasiliensis***	*2*	493, 494	-	-	-
***Vibrio breoganii***	*1*	1958	-	-	-
***Vibrio campbellii***	*3*	62, 416, 2415	-	-	-
***Vibrio chagasii***	*4*	583, 586, 640, 2353	-	-	-
***Vibrio cortegadensis***	*2*	529, 1974	-	-	-
***Vibrio gallaecicus***	*4*	528, 2315, 2319, 2327	-	-	-
***Vibrio gigantis***	*16*	503, 530, 853, 1230, 1232, 1233, 1972, 1973, 1980, 1982, 1989, 2045, 2312, 2357, 2372, 2412	-	-	-
***Vibrio harveyi***	*7*	558, 576, 579, 605, 615, 626, 2366	-	-	-
***Vibrio hemicentroti***	*2*	2269, 2311	-	-	-
***Vibrio ichthyoenteri***	*2*	490, 491	-	-	-
***Vibrio lentus***	*3*	495, 850, 851	-	-	-
***Vibrio maritimus***	*1*	2338	-	-	-
***Vibrio metschnikovii***	*2*	1026, 1055	-	+	+
***Vibrio mytili***	*1*	2428	+	-	-
***Vibrio natriegens***	*1*	546	-	-	-
***Vibrio neptunius***	*1*	496	-	-	-
***Vibrio ordalii***	*1*	1015	+	+	+
***Vibrio owensii***	*3*	1143, 1169, 1955	-	-	-
***Vibrio pectenicida***	*1*	1971	-	-	-
***Vibrio pomeroyi***	*2*	502, 1962	-	-	-
***Vibrio rumoiensis***	*2*	1210, 1211	-	-	-
***Vibrio scophtalmi***	*3*	1228, 1237, 1238	-	+	+
***Vibrio scophtalmi***	*4*	2361, 2365, 2370, 2413	-	-	-
***Vibrio shilonii***	*3*	2339, 2351, 2363	-	-	-
***Vibrio sinaloensis***	*1*	2347	-	-	-
***Vibrio splendidus***	*8*	66, 67, 165, 239, 498, 500, 527, 852	-	-	-
***Vibrio tasmaniensis***	*1*	526	-	-	-
***Vibrio tasmaniensis* LGP32**	*1*	2197	+	+	-
***Vibrio tubiashi***	*4*	620, 1231, 2159, 2190	-	-	-

**Table 2:** Limit of detection (nmol·L^−1^) of AHL standards using UHPLC-HRMS and three different biosensors: F117, *Pseudomonas putida* (pKR-C12); MT102, *Escherichia coli* (pJBA-132) and CV026, *Chromobacterium violaceum*. ND: not detected. MD: missing data.

	Limit of Detection (nmol·L^−1^)			
	CV026	MT102	F117	UHPLC-HRMS
**C4-HSL**	250	ND	ND	>500
**C6-HSL**	2.5	0.631	312.38	3.64
**OXO-C6-HSL**	10	<0.001	ND	10.90
**C7-HSL**	1	0.094	ND	5.33
**C8-HSL**	5	1.125	212.49	6.50
**OXO-C8-HSL**	10	0.0024	0.76	6.15
**OH-C8-HSL**	100	ND	7.077	MD
**C9-HSL**	5	1.93	2.89	7.37
**C10-HSL**	100	74.52	1.5	4.56
**OXO-C10-HSL**	1000	0.07	<0.001	2.91
**OH-C10-HSL**	2.5	ND	13.43	3.23
**C11-HSL**	ND	ND	<0.001	9.11
**C12-HSL**	ND	ND	0.01	5.07
**OXO-C12-HSL**	ND	0.702	<0.001	21.28
**OH-C12-HSL**	ND	ND	0.125	2.39
**C13-HSL**	ND	ND	0.00475	14.78
**C14-HSL**	ND	ND	0.608	11.82
**C14:1-HSL**	ND	0.366	0.00535	9.41
**OXO-C14-HSL**	ND	0.76	<0.001	6.79
**OXO-C14:1-HSL**	ND	ND	0.0606	4.71
**OH-C14-HSL**	ND	ND	0.492	36.58
**C15-HSL**	ND	ND	0.094	15.11
**C16-HSL**	ND	ND	12.01	16.30
**C16:1-HSL**	ND	0.1	0.023	14.68
**OXO-C16:1-HSL**	ND	ND	6.28	6.75
**C18-HSL**	ND	ND	ND	10.48
**C18:1-HSL**	ND	ND	7.8	28.56

**Table 3:** UHPLC-HRMS data and AHL identification in *V. tasmaniensis* LGP32. Rt: Retention time. Theoretical mass correspond to the pseudo-molecular ion [M + H]^+^.

Fractions	Rt (min)	Observed Mass	Molecular Formula	Delta ppm	Identification			
					Name	Molecular Formula	Molecular Weight	Theoretical Mass
LGP32_l	10.04	298.2014	C_16_H_28_NO_4_	−0.554	OXO-C12-HSL	C_16_H_27_NO_4_	297.1940	298.2012
LGP32_m	9.82	300.2166	C_16_H_30_NO_4_	2.182	OH-C12-HSL	C_16_H_29_NO_4_	299.2096	300.2169
LGP32_o	9.90	256.1914	C_14_H_26_NO_3_	2.810	C10-HSL	C_14_H_25_NO_3_	255.1834	256.1907
LGP32_p	10.50	310.2383	C_18_H_32_NO_3_	2.158	C14:1-HSL	C_18_H_31_NO_3_	309.2303	310.2376

**Table 4:** UHPLC-HRMS data of AHL Standards. Rt: Retention time. Theoretical mass correspond to the pseudo-molecular ion [M + H]^+^. MS/MS spectra for AHL standards can be found in Supplementary Information (Figure S1).

AHL Standard	Molecular Formula	Theorical Mass	Observed Mass	Rt (min)
**C4-HSL**	C_8_H_13_NO_3_	172.0968	172.0968	5.26
**C6-HSL**	C_10_H_17_NO_3_	200.1281	200.1281	8.43
**OXO-C6-HSL**	C_10_H_15_NO_3_	214.1074	214.1072	7.56
**C7-HSL**	C_11_H_19_NO_3_	214.1438	214.1440	8.83
**C8-HSL**	C_12_H_21_NO_3_	228.1594	228.1594	9.27
**OXO-C8-HSL**	C_12_H_19_NO_4_	242.1387	242.1381	8.69
**OH-C8-HSL**	C_12_H_21_NO_4_	244.1543	244.154	8.55
**C9-HSL**	C_13_H_23_NO_3_	242.1751	242.1748	9.57
**C10-HSL**	C_14_H_25_NO_3_	256.1907	256.1907	9.90
**OXO-C10-HSL**	C_14_H_23_NO_4_	270.1700	270.1699	9.43
**OH-C10-HSL**	C_14_H_25_NO_4_	272.1856	272.1856	9.25
**C11-HSL**	C_15_H_27_NO_3_	270.2064	270.2063	10.13
**C12-HSL**	C_16_H_29_NO_3_	284.2220	284.2220	10.46
**OXO-C12-HSL**	C_16_H_27_NO_4_	298.2013	298.2013	10.04
**OH-C12-HSL**	C_16_H_29_NO_4_	300.2169	300.2169	9.87
**C13-HSL**	C_17_H_31_NO_3_	298.2377	298.2377	10.63
**C14-HSL**	C_18_H_33_NO_3_	312.2533	312.2533	10.93
**C14:1-HSL**	C_18_H_31_NO_3_	310.2377	310.2370	10.51
**OXO-C14:1-HSL**	C_18_H_29_NO_4_	324.2169	324.2170	10.23
**OXO-C14-HSL**	C_18_H_31_NO_4_	326.2326	326.2322	10.56
**OH-C14-HSL**	C_18_H_33_NO_4_	328.2482	328.2482	10.42
**C15-HSL**	C_19_H_35_NO_3_	326.2690	326.2689	11.15
**C16-HSL**	C_20_H_37_NO_3_	340.2846	340.2846	11.34
**C16:1-HSL**	C_20_H_35_NO_3_	338.2690	338.2704	10.93
**OXO-C16:1-HSL**	C_20_H_33_NO_4_	352.2482	352.2497	10.61
**C18-HSL**	C_22_H_42_NO_3_	368.3159	368.3155	11.66
**C18:1-HSL**	C_22_H_39_NO_3_	366.3003	366.3003	11.40

## Supplementary Materials

The following are available online at http://www.mdpi.com/1424-8220/17/4/906/s1, Figure S1: Fragmentation MS/MS spectra of AHL standards, Figure S2: Maximum likelihood tree of 16S rDNA gene sequence (559 bp) of the 87 isolates and 35 type strains of *Vibrio* using the Kimura 2 parameter (K2+G+I, Mega), Table S1: List of the 87 tested strains with their origin, their identification by 16S rRNA gene sequence and their GenBank accession numbers.
