# Supplementary material for: Characterization of N-Acyl Homoserine Lactones in Vibrio tasmaniensis LGP32 by a Biosensor-Based UHPLC-HRMS/MS Method
Source: Sensors (Basel). 2017 Apr 20;17(4):906. doi: 10.3390/s17040906 (PMC5426830; doi:10.3390/s17040906)
Supplement: Supplementary file 1 [file sensors-17-00906-s001.pdf]

Supplementary material

Figure S1 : Fragmentation MS/MS spectra of AHL standards

C4-HSL

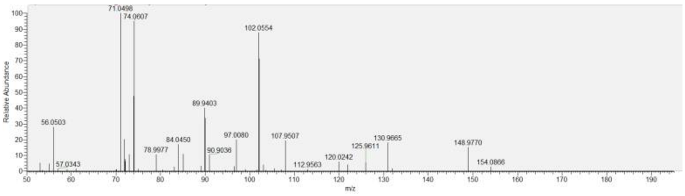

C6-HSL

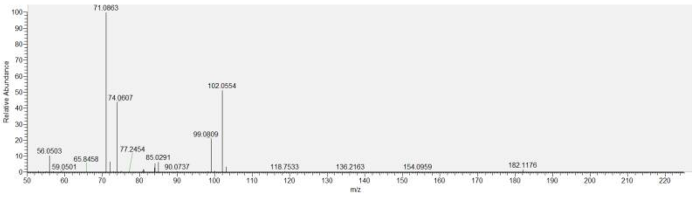

OXO-C6-HSL

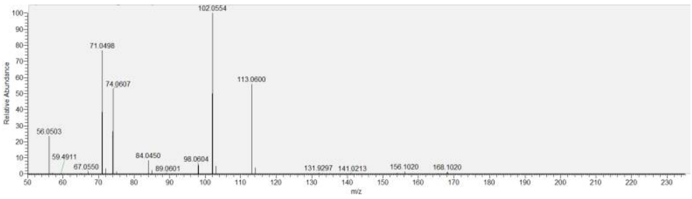

C7-HSL

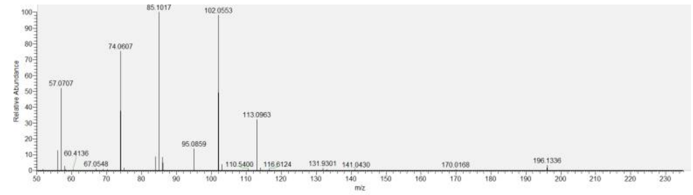

C8-HSL

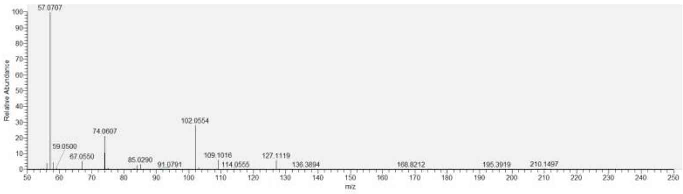

OXO-C8-HSL

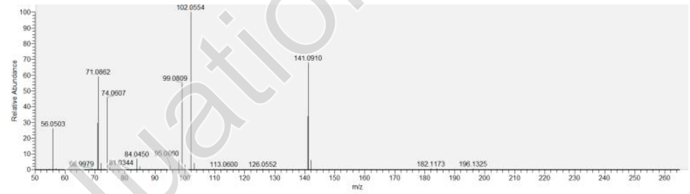

OH-C8-HSL

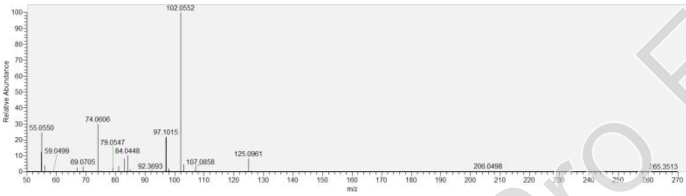

C9-HSL

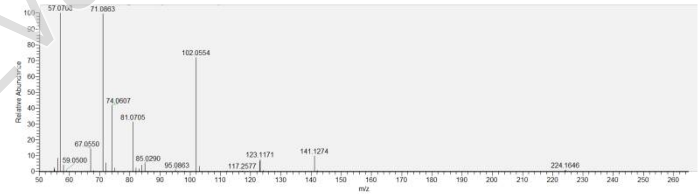

C10-HSL

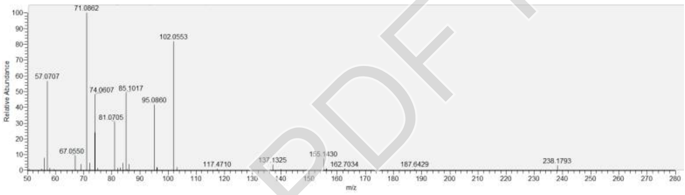

OXO-C10-HSL

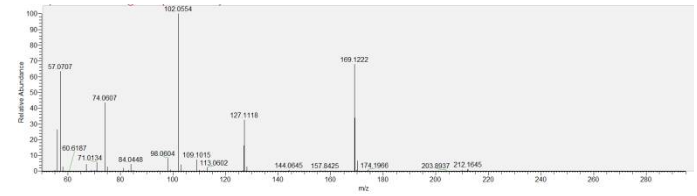

OH-C10-HSL

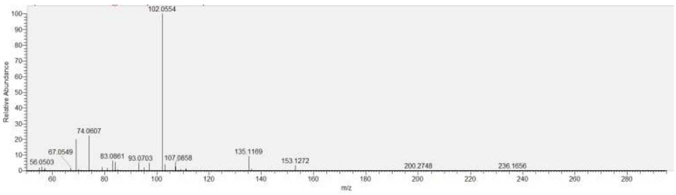

C11-HSL

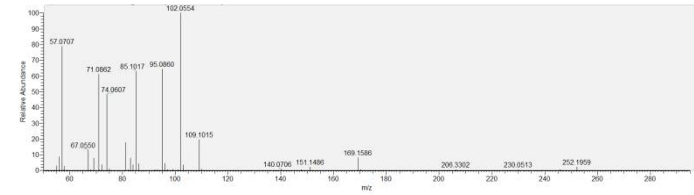

C12-HSL

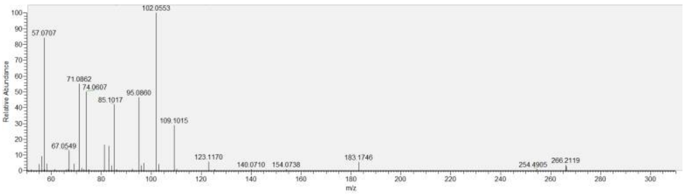

OXO-C12-HSL

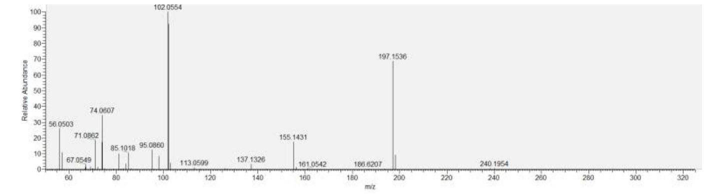

PDF Pro Evaluation

OH-C12-HSL

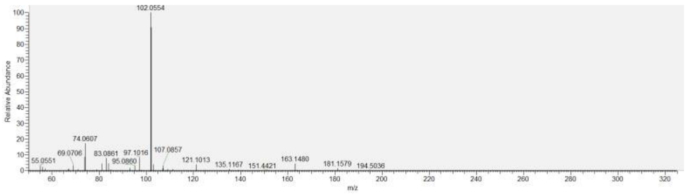

C13-HSL

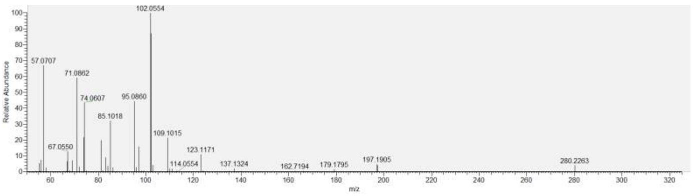

C14-HSL

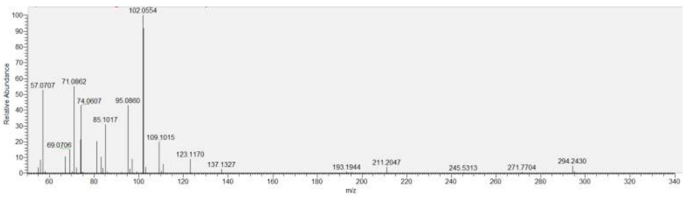

C14:1-HSL

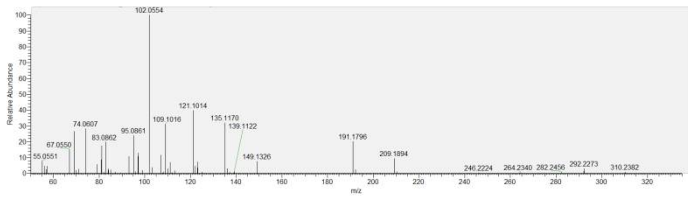

OXO-C14:1-HSL

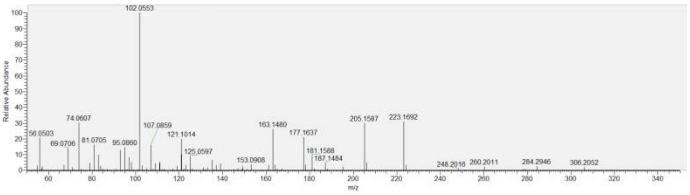

OXO-C14-HSL

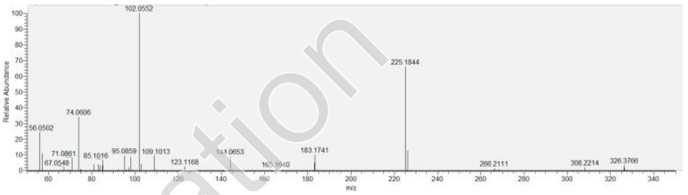

OH-C14-HSL

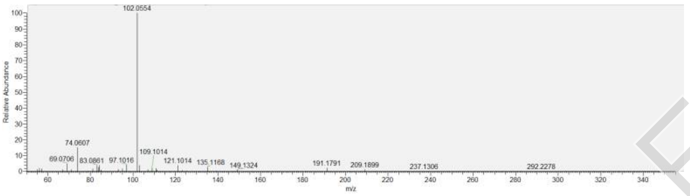

C15-HSL

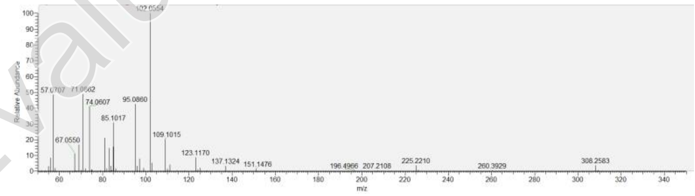

C16-HSL

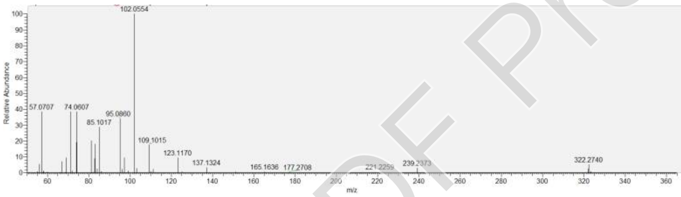

C16:1-HSL

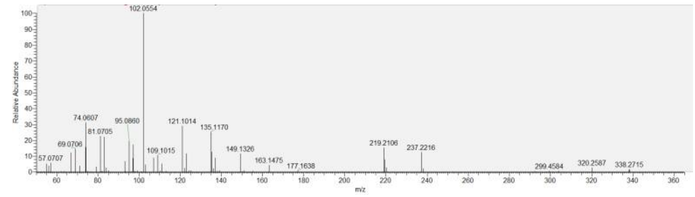

OXO-C16:1-HSL

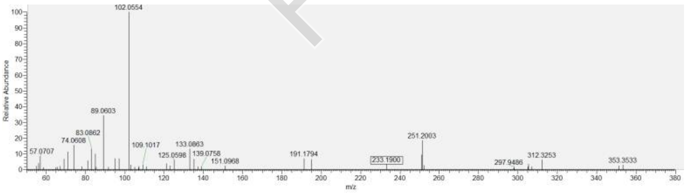

C18-HSL

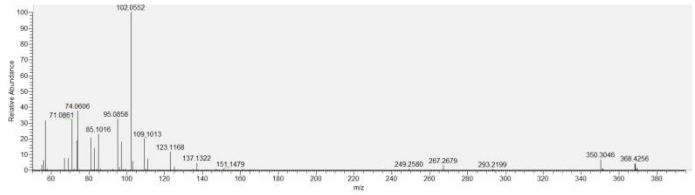

C18:1-HSL

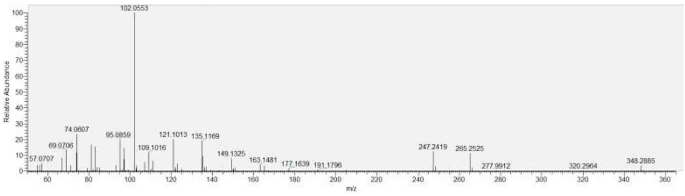

PDF Pro Evaluation

Figure S2 : Maximum likelihood tree of 16S rDNA gene sequence (559 bp) of the 87 isolates and 35 type strains of Vibrio using the Kimura 2 parameter (K2+G+I, Mega). Bootstrap values are based on 100 replicates. Black shapes are indicating AHL producing strains.

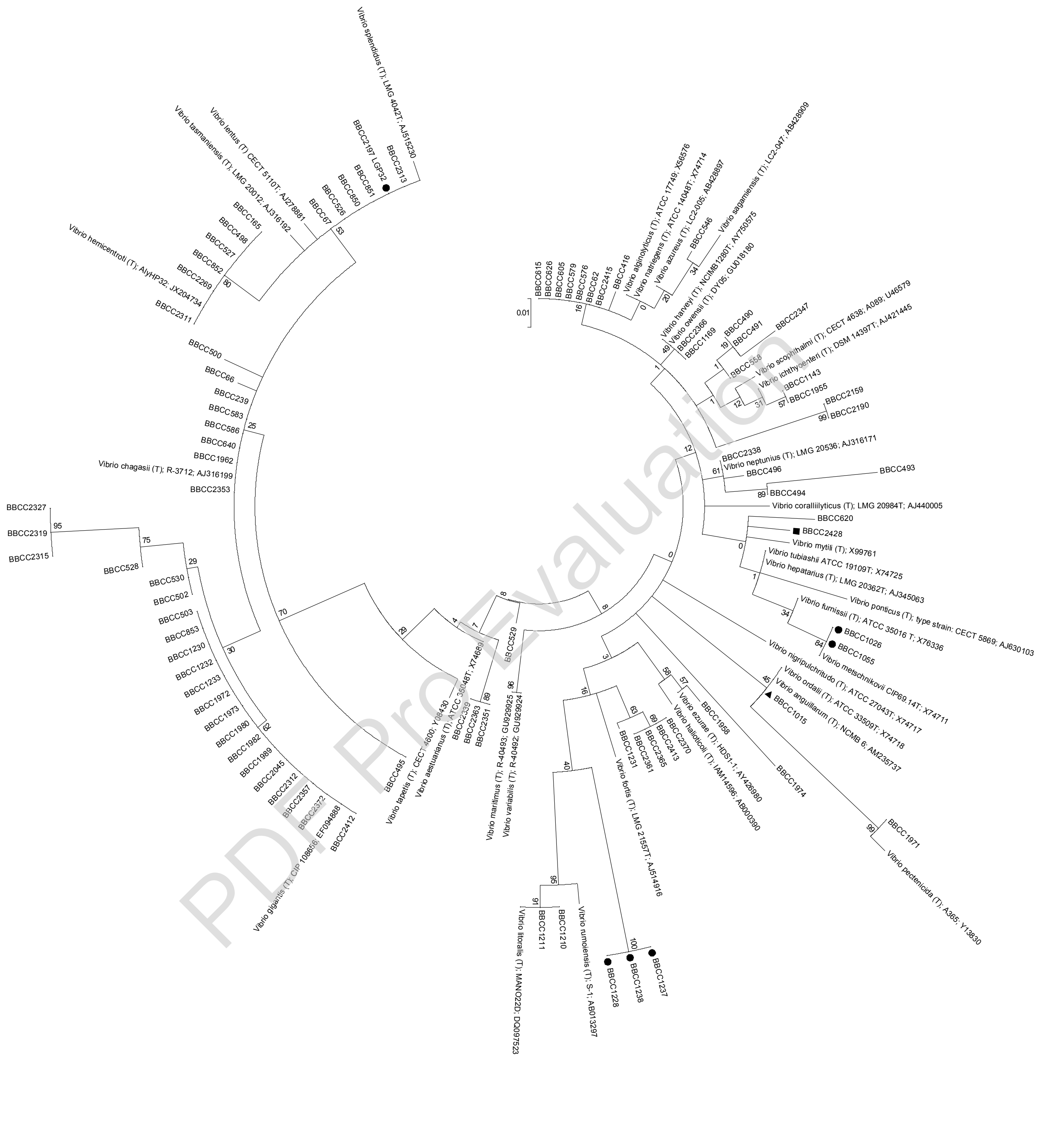

PDF Pro Evaluation

Table S1 : List of the 87 tested strains with their origin, their identification by 16S rRNA gene sequence and their GenBank accession numbers. SOLA, Banyuls Bay, Barcelona Bay, Leucate and Bages-Sigean Lagoons are located in the North-Western Mediterranean sea.

BBCC	Closest relative species	% of similarity	Origin	Accession number
**62**	*Vibrio campbellii*	99	SW, 3m, SOLA station	KY697620
**66**	*Vibrio splendidus*	99	SW, 3m, SOLA station	AM990841
**67**	*Vibrio splendidus*	99	SW, 3m, SOLA station	AM990842
**165**	*Vibrio splendidus*	99	SW, surface, Barcelona Bay, Spain	AY576700
**239**	*Vibrio splendidus*	99	SW, 3m, SOLA station	AY612761
**416**	*Vibrio campbellii*	99	SW, surface, Guanabara Bay, Brazil	AM990683
**490**	*Vibrio ichthyoenteri*	99	SW, 3m, SOLA station	KY697621
**491**	*Vibrio ichthyoenteri*	99	SW, 3m, SOLA station	KY697622
**493**	*Vibrio brasiliensis*	99	SW, 3m, SOLA station	KY697623
**494**	*Vibrio brasiliensis*	99	SW, 3m, SOLA station	KY697624
**495**	*Vibrio lentus*	100	SW, 3m, SOLA station	KY697625
**496**	*Vibrio neptunius*	99	SW, 3m, SOLA station	KY697626
**498**	*Vibrio splendidus*	99	*Petrosia ficiformis,* Banyuls Bay	AM990723
**500**	*Vibrio splendidus*	99	*Petrosia ficiformis,* Banyuls Bay	AM990725
**502**	*Vibrio pomeroyi*	99	*Petrosia ficiformis,* Banyuls Bay	AM990727
**503**	*Vibrio gigantis*	99	*Petrosia ficiformis,* Banyuls Bay	AM990728
**526**	*Vibrio tasmaniensis*	99	*Petrosia ficiformis,* Banyuls Bay	AM990750
**527**	*Vibrio splendidus*	99	*Petrosia ficiformis,* Banyuls Bay	AM990751
**528**	*Vibrio gallaecicus*	100	*Petrosia ficiformis,* Banyuls Bay	AM990752
**529**	*Vibrio cortegadensis*	100	*Petrosia ficiformis,* Banyuls Bay	AM990753
**530**	*Vibrio gigantis*	99	*Petrosia ficiformis,* Banyuls Bay	AM990754
**546**	*Vibrio natriegens*	99	SW, water column, Po Delta, Italy	KY697627
**558**	*Vibrio harveyi*	99	SW, water column, Po Delta, Italy	KY697628
**576**	*Vibrio harveyi*	100	SW, water column, Po Delta, Italy	KY697629
**579**	*Vibrio harveyi*	100	SW, water column, Po Delta, Italy	KY697630
**583**	*Vibrio chagasii*	100	SW, water column, Po Delta, Italy	KY697631
**586**	*Vibrio chagasii*	100	SW, water column, Po Delta, Italy	KY697632
**605**	*Vibrio harveyi*	100	SW, water column, Po Delta, Italy	KY697633
**615**	*Vibrio harveyi*	100	SW, water column, Po Delta, Italy	KY697634
**620**	*Vibrio tubiashi*	99	SW, water column, Po Delta, Italy	KY697635
**626**	*Vibrio harveyi*	100	SW, water column, Po Delta, Italy	KY697636
**640**	*Vibrio chagasii*	100	SW, water column, Po Delta, Italy	KY697637
**850**	*Vibrio lentus*	99	SW, surface, Bages-Sigean Lagoon	KY697638
**851**	*Vibrio lentus*	99	SW, surface, Bages-Sigean Lagoon	KY697639
**852**	*Vibrio splendidus*	99	SW, surface, Bages-Sigean Lagoon	KY697640
**853**	*Vibrio gigantis*	99	SW, surface, Bages-Sigean Lagoon	KY697641
**1015**	*Vibrio ordalii*	100	SW, surface, Bages-Sigean Lagoon	KY697642
**1026**	*Vibrio metschnikovii*	100	Dziani Dzaha halo alkali crater lake, Mayotte	KX818008
**1055**	*Vibrio metschnikovii*	100	Dziani Dzaha halo alkali crater lake, Mayotte	KX818036
**1143**	*Vibrio owensii*	99	SW, surface, Marsa alam (Red sea)	KY697643
**1169**	*Vibrio owensii*	100	SW, surface, Marsa alam (Red sea)	KY697644
**1210**	*Vibrio rumoiensis*	99	SW, surface, Canet Lagoon	KY697645
**1211**	*Vibrio rumoiensis*	99	SW, Surface, Canet Lagoon	KY697646
**1228**	*Vibrio scophtalmi*	98	Diseased *Paracentrotus lividus,* Banyuls Bay	KY697647
**1230**	*Vibrio gigantis*	100	Healthy *Paracentrotus lividus,* Banyuls Bay	KY697648

PDF Pro Evaluation

**1231**	*Vibrio tubiashi*	99	Healthy *Paracentrotus lividus,* Banyuls Bay	KY697649
**1232**	*Vibrio gigantis*	100	Healthy *Paracentrotus lividus,* Banyuls Bay	KY697650
**1233**	*Vibrio gigantis*	100	Healthy *Paracentrotus lividus,* Banyuls Bay	KY697651
**1237**	*Vibrio scophtalmi*	98	Diseased *Paracentrotus lividus,* Banyuls Bay	KY697652
**1238**	*Vibrio scophtalmi*	98	Diseased *Paracentrotus lividus,* Banyuls Bay	KY697653
**1955**	*Vibrio owensii*	99	*Ulva sp*., Banyuls Bay	KY697654
**1958**	*Vibrio breoganii*	99	*Ulva sp*., Banyuls Bay	KY697655
**1962**	*Vibrio pomeroyi*	100	SW, Banyuls Bay	KY697656
**1971**	*Vibrio pectenicida*	99	SW, Banyuls Bay	KY697657
**1972**	*Vibrio gigantis*	100	SW, Banyuls Bay	KY697658
**1973**	*Vibrio gigantis*	100	SW, Banyuls Bay	KY697659
**1974**	*Vibrio cortegadensis*	99	SW, Banyuls Bay	KY697660
**1980**	*Vibrio gigantis*	100	*Ulva sp*., Banyuls Bay	KY697661
**1982**	*Vibrio gigantis*	100	*Ulva sp*., Banyuls Bay	KY697662
**1989**	*Vibrio gigantis*	100	*Ulva sp*., Banyuls Bay	KY697663
**2045**	*Vibrio gigantis*	99	*Peyssonnelia sp*., Banyuls Bay	KY697664
**2159**	*Vibrio tubiashi*	99	*Posidonia oceanica*, Banyuls Bay	KY697665
**2190**	*Vibrio tubiashi*	99	*Posidonia oceanica*, Banyuls Bay	KY697666
**2197**	*Vibrio tasmaniensis LGP32*		CIP 107715, Diseased oyster *C. gigas*	NC_011753
**2269**	*Vibrio hemicentroti*	99	SW, 24m, SOLA station	KY697667
**2311**	*Vibrio hemicentroti*	99	*Salpa sp.*, Banyuls Bay	KY697668
**2312**	*Vibrio gigantis*	100	*Salpa sp.*, Banyuls Bay	KY697669
**2313**	*Vibrio atlanticus*	99	*Salpa sp.*, Banyuls Bay	KY697670
**2315**	*Vibrio gallaecicus*	99	*Salpa sp.*, Banyuls Bay	KY697671
**2319**	*Vibrio gallaecicus*	99	*Salpa sp.*, Banyuls Bay	KY697672
**2327**	*Vibrio gallaecicus*	99	*Salpa sp.*, Banyuls Bay	KY697673
**2338**	*Vibrio maritimus*	99	*Rhizostoma pulmo*, Leucate Lagoon	KY697674
**2339**	*Vibrio shilonii*	99	*Rhizostoma pulmo*, Leucate Lagoon	KY697675
**2347**	*Vibrio sinaloensis*	99	*Corticium candelabrum,* Banyuls Bay	KY697676
**2351**	*Vibrio shilonii*	99	*Corticium candelabrum,* Banyuls Bay	KY697677
**2353**	*Vibrio chagasii*	100	*Corticium candelabrum,* Banyuls Bay	KY697678
**2357**	*Vibrio gigantis*	100	*Corticium candelabrum,* Banyuls Bay	KY697679
**2361**	*Vibrio scophtalmi*	99	*Corticium candelabrum,* Banyuls Bay	KY697680
**2363**	*Vibrio shilonii*	99	*Corticium candelabrum,* Banyuls Bay	KY697681
**2365**	*Vibrio scophtalmi*	99	*Corticium candelabrum,* Banyuls Bay	KY697682
**2366**	*Vibrio harveyi*	99	*Corticium candelabrum,* Banyuls Bay	KY697683
**2370**	*Vibrio scophtalmi*	99	*Corticium candelabrum,* Banyuls Bay	KY697684
**2372**	*Vibrio gigantis*	100	*Corticium candelabrum,* Banyuls Bay	KY697685
**2412**	*Vibrio gigantis*	100	*Corticium candelabrum,* Banyuls Bay	KY697686
**2413**	*Vibrio scophtalmi*	99	*Corticium candelabrum,* Banyuls Bay	KY697687
**2415**	*Vibrio campbellii*	100	*Corticium candelabrum,* Banyuls Bay	KY697688
**2428**	*Vibrio mytili*	99	*Corticium candelabrum,* Banyuls Bay	KY697689

PDF Pro Evaluation
